# Insertional mutagenesis enables cleistothecial formation in a non-mating strain of *Histoplasma capsulatum*

**DOI:** 10.1186/1471-2180-10-49

**Published:** 2010-02-16

**Authors:** Meggan C Laskowski, Alan G Smulian

**Affiliations:** 1 Infectious Disease Division, University of Cincinnati College of Medicine, 231 Albert Sabin Way, Cincinnati, OH 45267-0560, USA; 2 Department of Pathology, University of Cincinnati College of Medicine, 231 Albert Sabin Way, Cincinnati, OH 45267, USA; 3 Cincinnati VA Medical Center, 3200 Vine Street, Cincinnati, OH 45220, USA

## Abstract

**Background:**

*Histoplasma capsulatum *is a pathogenic ascomycete fungus that rapidly loses mating ability in culture. Loss of mating ability, as well as the organism's low rate of targeted gene replacement, limits techniques available for genetic studies in *H. capsulatum*. Understanding molecular mechanisms regulating mating in this organism may allow us to reverse or prevent loss of mating in *H. capsulatum *strains, introducing a variety of classical genetics techniques to the field. We generated a strain, UC1, by insertional mutagenesis of the laboratory strain G217B, and found that UC1 acquired the ability to form mating structures called cleistothecia. The aim of this study was to determine the mechanism by which UC1 gained the ability to form cleistothecia. We also present initial studies demonstrating that UC1 can be used as a tool to determine molecular correlates of mating in *H. capsulatum*.

**Results:**

The strain UC1 was found to have increased RNA levels of the mating locus transcription factor (*MAT1-1-1*), and the putative alpha pheromone (*PPG1*) compared to G217B. *Agrobacterium*-mediated transformation and integration of T-DNA from the vector pCB301-GFP-HYG were found to be partially responsible for the increased RNA levels of these genes; however, the site of integration appeared to play the largest role in the strain's ability to form cleistothecia. Silencing *HMK1*, a putative *FUS3/KSS1 *homolog, had no effect on cleistothecial production by UC1. Protein kinase C *(PKC1) *RNA and protein levels were increased in UC1 compared to G217B, and pheromone production was found to be linked with Pkc1 activity in *H. capsulatum*.

**Conclusions:**

The site of the T-DNA integration event appears to play the largest role in UC1's ability to form cleistothecia. We show that the UC1 strain can be used as a tool to study cleistothecia production in *H. capsulatum *by manipulating the strain, or by identifying differences between UC1 and G217B. Using these approaches, we were able to link Pkc1 activity with pheromone production in *H. capsulatum*; however, further studies are required to determine molecular mechanisms behind this. These studies may reveal regulatory mechanisms that can be manipulated to restore mating ability in *H. capsulatum *laboratory strains.

## Background

*Histoplasma capsulatum *is a heterothallic ascomycete fungus requiring two mating types, phenotypically described as (+) and (-), to undergo mating [1]. Organisms of (+) mating type have the *MAT1-1 *idiomorph of the mating locus, while organisms of (-) mating type have the *MAT1-2 *idiomorph of the mating locus [2]. The fungus is pathogenic, and organisms of (-) mating type may be associated with increased virulence. The organism causes acute pulmonary disease when inhaled into the lung [3-5]. Individuals may also develop the disseminated form of the disease, which is usually controlled by activation of cell-mediated immunity in immune-competent individuals [4,6]. Organisms of (-) mating type are found more frequently in samples from patients with pulmonary histoplasmosis; however, organisms of both mating types are represented equally in samples from patients with severe disseminated histoplasmosis and in environmental samples [7,8]. It is unknown whether the (-) mating type strain predominates in clinical samples due to host factors, or differences between organisms of opposite mating type. A single study examining virulence of (+) and (-) mating type strains has been reported; however, interpretation is limited by the inability to compare congenic strains of *H. capsulatum *[9]. Mating occurs under appropriate conditions in the mycelial phase when hyphae arising from organisms of opposite mating type appose and generate a complex structure comprising of a net of short branching hyphae covered with coiled surface hyphae. Within this specialized closed structure, the cleistothecium, cytoplasmic and nuclear fusion occur followed by successive rounds of meiosis and mitosis generating sac-like asci containing 8 ascospores, the end-product of sexual replication.

Generation of congenic strains in *H. capsulatum *is challenging due to the low frequency of homologous gene targeting in the organism [10], and because the organism rapidly loses mating ability in culture [7]. This limits the feasibility of gene replacement or backcrossing as methods for generating congenic strains. If the loss of mating competency could be overcome in laboratory strains of *H. capsulatum*, a variety of classical genetics techniques could be developed for use in this organism, including congenic strain construction. Understanding the molecular mechanisms that regulate mating could lead to the restoration of mating ability in laboratory strains of *H. capsulatum*. Through this work, we generated a strain of *H. capsulatum *that can be used to examine molecular correlates of mating.

Regulation of mating in fungi requires integration of multiple pathways in a complex developmental program. The pheromone response MAP kinase pathway is a central pathway in the mating response of many fungi [11]. In the model fungus *Saccharomyces cerevisiae*, this pathway allows yeasts to sense a mating partner, and coordinates appropriate responses such as G1 arrest [12,13]. MAP kinase pathways homologous to the pheromone response MAP kinase pathway have been identified in several fungi including *Candida albicans *[14,15], *Neurospora crassa *[16], and *Paracoccidiodes brasiliensis *[17]. There is also evidence in *S. cerevisiae *for a functional link between the pheromone response MAP kinase pathway and the MAP kinase pathway involved in cell wall integrity, as *S. cerevisiae *strains lacking the MAP kinase Slt2 die after exposure to pheromone [18]. Transcription factors present at the mating locus are additional regulators of mating in fungi such as *Cryptococcus neoformans *and *C. albicans *[19,20]. The *MAT1-1-1 *and *MAT1-2-1 *transcription factors of *H. capsulatum *have previously been shown to be transcriptionally responsive to conditioned media enriched for pheromone [2], indicating that these transcription factors play a role in the mating process of *H. capsulatum *as well.

We generated a laboratory strain, UC1, which was capable of forming empty cleistothecia when paired with a fresh clinical strain of opposite mating type. Unlike other strains of *H. capsulatum*, UC1 did not lose the ability to form cleistothecia over time. We hypothesized that understanding how UC1 gained the ability to form cleistothecia would explain how *H. capsulatum *strains lose the ability to mate over time. We sought to determine how UC1 gained the ability to form cleistothecia, and then determined that UC1 could be used to identify molecular events contributing to cleistothecia production in *H. capsulatum*. *H. capsulatum *is a dimorphic fungus, growing in the yeast phase at 37°C and in mycelial phase at room temperature. Because mating occurs in the mycelial phase, these studies were performed using organisms growing in the mycelial phase.

The UC1 strain was originally generated by *Agrobacterium tumefaciens*-mediated transformation and integration of the T-DNA region from the vector pCB301-GFP-HYG into the strain G217B [21]. The strain G217B was isolated in 1973 [22], has been extensively passaged in the laboratory, and is itself unable to form cleistothecia. The UC1 strain, derived by transformation of the G217B strain, is thought to have gained the ability to produce empty cleistothecia due to a combination of the transformation procedure itself, and the site of T-DNA integration. We used the UC1 strain to study cleistothecia formation by searching for differences between UC1 and its parent G217B, and we determined that the *H. capsulatum *homolog of protein kinase C (*PKC1*) plays a role in cleistothecia formation.

## Results

### Characterization of cleistothecia-like structures formed by UC1 and UH3

**Figure 1:**
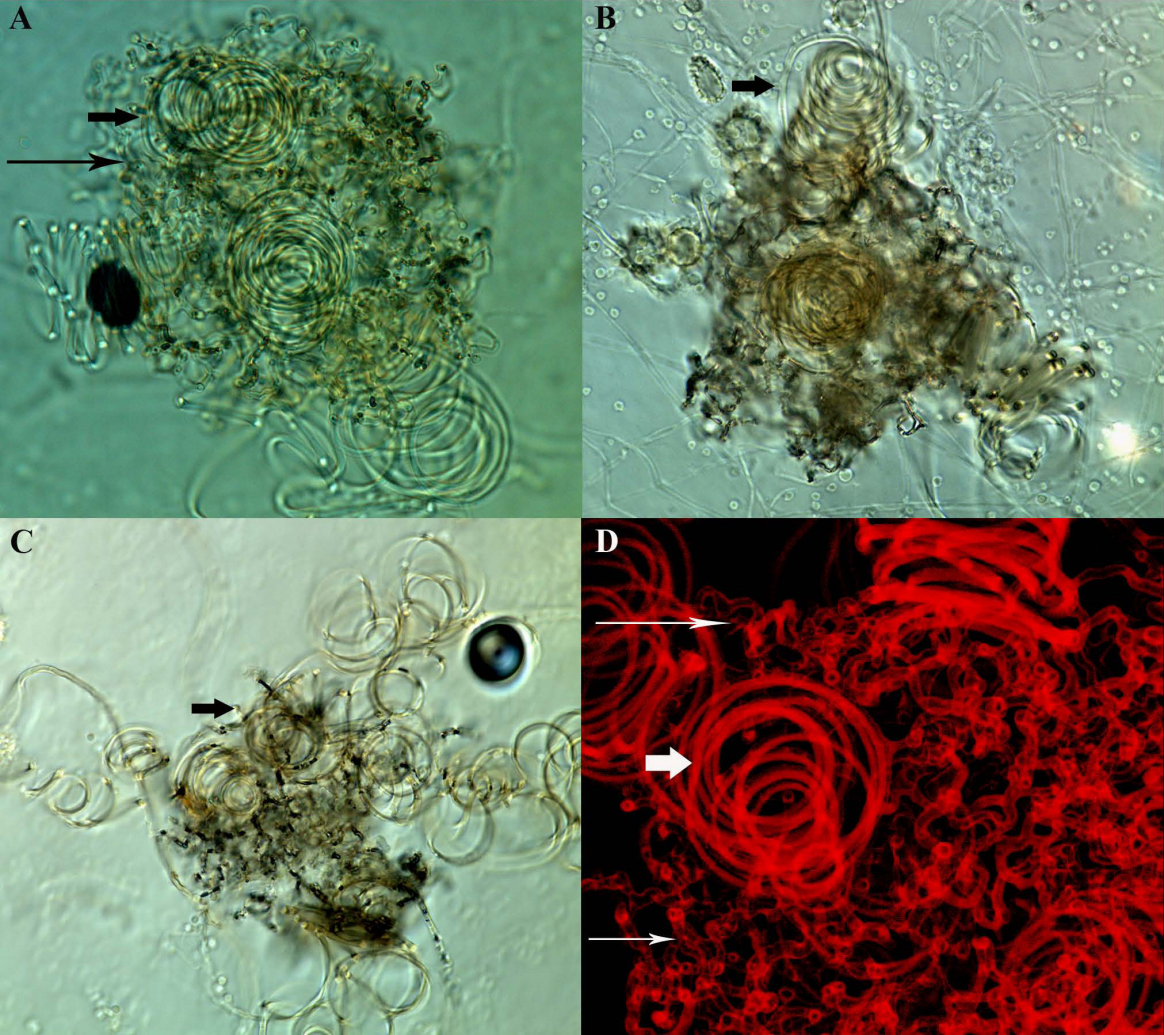
**Cleistothecia formed by mating crosses**. A: Cleistothecia formed by UH3 and UC1, DIC image, 400×. B: Cleistothecia formed by UH3 and UC26, DIC image, 400×. C: Dissected cleistothecia from UH3 and UC26 pairing, DIC image, 400×. D: Alpha projection of Z-stack taken of cleistothecia formed by UH3 and UC1, confocal image of autofluorescence, 600×. The coiled surface hyphae are identified by short arrows while the net of short, branched hyphae are identified by long arrows.

**Figure 2:**
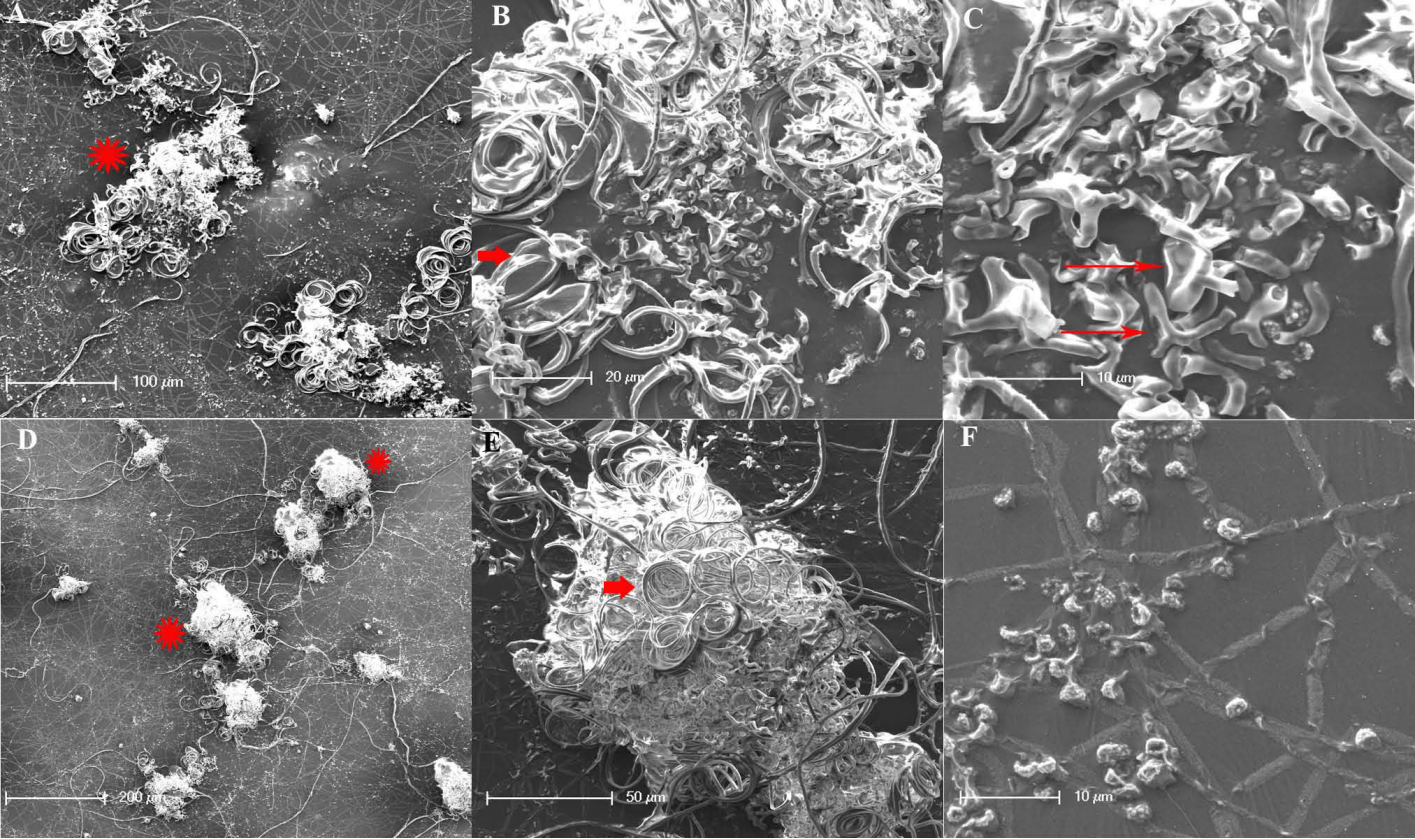
**SEM images of cleistothecia formed by UH3 and UC1**. A: Dissected cleistothecia, 200×. B: View A, 1000×. C: View B, 2500×. D: Whole cleistothecia, 100×. E: View D, 500×. F: Microconidia, 2000×. In panels A and D, cleistothecia are identified by symbol *, while coiled surface hyphae are identified by short arrows while the net of short, branched hyphae are identified by long arrows where appropriate.

The strain UC1 formed cleistothecia when paired with the fresh clinical strain UH3. Cleistothecia were visible to the naked eye at the periphery of the colony when mycelial scrapings of each strain were co-incubated on A-YEM agarose at room temperature for one month. At 400-500×, the net-like hyphae forming the cleistothecia were visible, as were characteristic coiling hyphae radiating from the cleistothecia (Figure 1A, Figure 2E). These structures were not observed when UH3 was crossed with G217B, the parent strain of UC1. UC1 formed cleistothecia-like structures in greater than 90% of confrontation assays within 6 weeks when paired with Mat1-2 clinical strains passaged for less than 6 months. UC1 maintained the ability to form cleistothecia for more than 4 years after generation of the strain from strain G217B. No cleistothecia were formed when UH3 and UC1 were paired with UH1 and VA1, respectively, two clinical strains that had been passaged for several months in the laboratory, consistent with previous reports that loss of mating competence occurred after 5-8 months of continuous passage. The exact timing of the loss of mating competence of *H. capsulatum *G217B is unknown as the strain was first reported in 1973 and has been maintained in culture since then. Nutrient limiting media was required for cleistothecia formation, as UC1 and UH3 did not form cleistothecia on nutrient-rich HMM.

Cleistothecia were partially dissected to determine whether asci, containing ascospores, had been produced by the crosses. The cleistothecia appeared empty, as no clusters of club-shaped asci were visible by light microscopy (Figure 1C) or scanning electron microscopy (SEM) (Figure 2A-C) when structures were teased apart prior to visualization. Only what appear to be microconidia were observed by SEM when cleistothecia-like structures were dissected (Figure 2C, F). Alpha projections of Z-stacks taken by confocal microscopy also showed no evidence of asci (Figure 1D). Additionally UH3-Blast, a blasticidin resistant strain of UH3 was generated and crossed with UC1. Cleistothecia from this cross were dissected and transferred to plates containing hygromycin and blasticidin, where no growth was observed after several weeks. These results indicate that while the strain UC1 can form empty cleistothecia, it is unable to complete the mating process by producing asci and ascospores.

### Molecular comparison of UC1 and G217B

**Figure 3:**
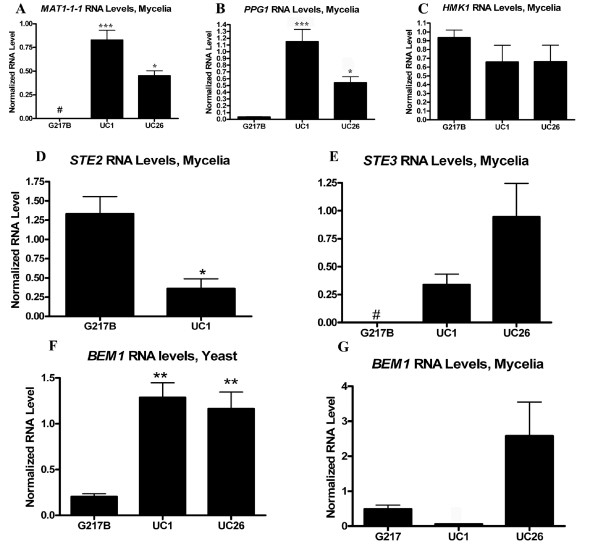
**Molecular differences between G217B, UC1, and UC26**. A-C: *MAT1-1-1*, *PPG1*, and *HMK1 *RNA levels in G217B, UC1, and UC26 mycelial samples as measured by qRT-PCR. D, E: *STE2 *and *STE3 *RNA levels in G217B and UC1 mycelial samples, measured by qRT-PCRr. F, G: *BEM*1 RNA levels in G217B, UC1, and UC26 yeast (F) and mycelial (G) samples, measured by qRT-PCR. Values represent the average and standard error of quadruplicate samples except 3A: UC1, n = 6; UC26, n = 4; 3D: UC1 n = 3; 3F: G217B & UC1, n = 3; 3G: n = 3. * = p ≤ 0.05 ** = p ≤ 0.01 *** = p ≤ 0.001 # = below level of detection.

To determine molecular correlates of the ability to form cleistothecia, RNA levels of gene products predicted to be involved in mating (Table 1) were analyzed in UC1 and G217B. Differences in expression of these genes could suggest the mechanism behind UC1's ability to form empty cleistothecia. Genes analyzed included the mating locus transcription factor *MAT1-1-1*[2], a putative alpha pheromone (*PPG1*, manuscript in preparation), and a putative Fus3/Kss1 homolog, Histoplasma Map Kinase-1 (*HMK1*). RNA levels of *MAT1-1-1 *were undetectable in mycelial samples of G217B, but were elevated in UC1 (Figure 3A). RNA levels of *PPG1 *were also elevated in UC1 compared to G217B (Figure 3B). In contrast, RNA levels of *HMK1 *were similar in UC1 and G217B (Figure 3C). RNA levels of *STE2 *and *STE3*, putative alpha and a pheromone receptors respectively, were also analyzed in UC1 and G217B. *STE2 *and *STE3 *were detectable in mycelial samples of UC1, while only *STE2 *was detectable in mycelial samples of G217B (Figure 3E, D). These results indicated that higher levels of *MAT1-1-1 *and *PPG1 *as well as differences in expression of pheromone receptors might contribute to the ability of UC1 to form empty cleistothecia.

**Table 1:** *H. capsulatum *genes predicted to be involved in mating

	**Identity with *S. cerevisiae *homolog**	**G217B gene alias**[42]** (gene name**[43])	**Nam1 gene name**[44]
	
***HMK1***	Fus3: 60.3% Kss1: 62.9%	HISTO_ZT.Contig1089.eannot.1595.final_new (HCB06569.1)	HCAG_05250.1
***STE2***	20.7%	HISTO_BP.Contig459.eannot.1558.final_new (HCB00638.1)	HCAG_01152
***STE3***	29%	HISTO_ZU.Contig65.Fgenesh_histo.124.final_new (HCB07122.1)	HCAG_02974
***BEM1***	35.9%	HISTO_FX.Contig167.Fgenesh_histo.29.final_new (HCB02453.1)	HCAG_08014
***PKC1***	44.4%	HISTO_LF.Contig359.Fgenesh_histo.161.final_new (HCB09506.1)	HCAG_02636

### Contribution of hygromycin phosphotransferase to cleistothecial formation

A series of experiments were performed to determine why RNA levels of genes involved in mating were increased in UC1, and to determine whether this had caused the strain's ability to form empty cleistothecia. The strain UC1 was generated by integrating T-DNA from the vector pCB301-GFP-HYG into the genome of the strain G217B by *Agrobacterium tumefaciens*-mediated transformation [21]. UC1 could have gained the ability to produce empty cleistothecia due to the site of T-DNA integration, or due to elements present within the T-DNA region itself. The effects of hygromycin phosphotransferase (*hph*) gene expression from within the T-DNA region were investigated first, because hph activity has previously been associated with increased virulence in the strain UC1 [21]. To determine whether *hph *expression was responsible for cleistothecia production by UC1, cleistothecia production was tested using the strain UC26. UC26 is a derivative of UC1 in which the *hph *gene has been excised from the integrated T-DNA region by Cre-mediated recombination [21]. RNA levels of *MAT1-1-1 *and *PPG1 *were still increased in UC26 compared to G217B (Figure 3A, B). UC26 also still formed empty cleistothecia when paired with UH3 (Figure 1B), indicating that *hph *expression is not necessary for cleistothecia production by UC1.

### Effects of T-DNA insertion on genes flanking site of integration

Expression patterns of genes flanking the site of T-DNA integration may have been altered in UC1 due to the insertion, and this might be responsible for the differences between UC1 and G217B. Effects of the site of T-DNA integration were analyzed in UC1 to investigate the cause of the differences between UC1 and G217B. It has previously been determined that the T-DNA is integrated upstream of HCAG 08014 in the strain UC1 [21]. HCAG 08014 shares sequence similarity with the *S. cerevisiae *Bem1 protein, a scaffold protein involved in polarity and also in the pheromone response MAP kinase pathway. RNA levels of putative Bem1 and of HCAG 08015, the two genes flanking the site of T-DNA integration, were analyzed. RNA levels of HCAG 08015 were undetectable in G217B and in UC1. RNA levels of *BEM1 *were increased in yeast phase UC1 and UC26 compared to G217B (Figure 3F). In mycelial phase organisms, when cleistothecia formation occurs, levels of *BEM1 *were increased in UC26 compared to G217B, but decreased in UC1 compared to G217B (Figure 3G). These results indicate that expression of the genes immediately flanking the T-DNA insertion site is not likely to be responsible for the ability of UC1 and UC26 to form empty cleistothecia.

### Effects of T-DNA insertion site on cleistothecia formation

To further explore the contribution of the site of T-DNA integration to the ability of UC1 to form cleistothecia, additional strains were generated with the same T-DNA sequence integrated elsewhere in the genome. If the site of T-DNA integration plays a major role in UC1's ability to form empty cleistothecia, then strains with the same T-DNA region integrated elsewhere in the genome would not be expected to form cleistothecia. If elements present within the T-DNA region are responsible for UC1's ability to form empty cleistothecia, then strains with the same T-DNA region integrated elsewhere in the genome would still be able to form cleistothecia.

**Figure 4:**
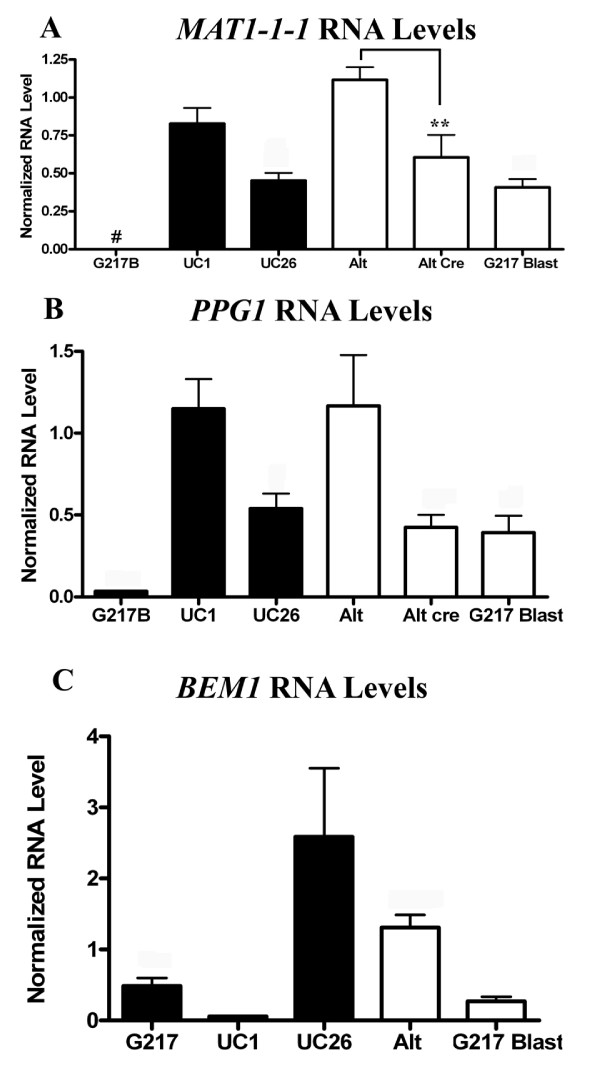
**Effects of T-DNA insertion from two different vectors on RNA levels of *MAT1-1-1*, *PPG1 *and *BEM1***. Comparison of G217B, UC1, and UC26 with strains with pCB301-HYG-GFP integrated at alternate sites (Alt), ALT strains with hph excised (Alt cre), or strains with pCB301-Blast integrated into the genome (G217B Blast). RNA levels of *MAT1-1-1 *(A), *PPG1 *(B), and *BEM1 *(C) in mycelial samples were compared by qRT-PCR. Alt samples represent the average of values obtained from triplicate samples of 4 different strains. Alt cre and G217 Blast samples represent the average of values obtained from triplicate samples of two different strains. n = 3 except 4A: UC1, n = 6; UC26, n = 4; 4B: n = 4 for G217B, UC1, and UC26. ** = p ≤ 0.01 # = below level of detection.

To distinguish effects of the site of T-DNA integration on cleistothecia production from effects due to elements present within the T-DNA region itself, four additional strains were generated in the G217B background: ALT8, ALT13, ALT15, and ALT16. These were generated by random integration of T-DNA from the same vector, pCB301-GFP-HYG, into alternative locations in the genome of G217B by *Agrobacterium*-mediated transformation. The site of *Agrobacterium*-mediated integration has previously been shown to be random in *H. capsulatum *[21,23,24]. RNA levels of *MAT1-1-1*, *PPG1*, and *BEM1 *were analyzed in these strains and compared to those of G217B, UC1, and UC26. RNA levels of *MAT1-1-1 *and *PPG1 *in strains ALT8, 13, 15, and 16 were comparable to those of UC1 (Figure 4A, B). However, the strains ALT8, 13, 15, and 16 were unable to produce cleistothecia when paired with UH3. These results indicate that the site of integration may play a role in the ability of UC1 and UC26 to form empty cleistothecia. This effect is independent of the increased *MAT1-1-1 *and *PPG1 *RNA levels in these strains, which may be due to elements within the T-DNA region or to the *Agrobacterium *transformation process itself.

### Effects of *hph *expression on *MAT1-1-1 *and *PPG1 *RNA levels

While *hph *expression is not necessary for empty cleistothecia production by UC1, it could be responsible for the increased RNA levels of *MAT1-1-1 *and *PPG1 *observed in strains that contain the *hph *gene within the T-DNA region. To determine the effects of *hph *on RNA levels of *MAT1-1-1 *and *PPG1 *in the strain ALT16, *hph *was excised from the integrated T-DNA region in this strain by Cre-mediated recombination. *MAT1-1-1 *and *PPG1 *RNA levels were decreased in the two Cre strains tested compared to UC1 and the original ALT16 strain (Figure 4A, B). This indicates that the increase in *MAT1-1-1 *and *PPG1 *RNA levels is partly due to the presence of *hph *in the integrated T-DNA region; however, this is not sufficient to induce cleistothecia production in the ALT strains.

### Effects of *Agrobacterium*-mediated transformation on *MAT1-1-1 *and *PPG1 *RNA levels

Since integration of the T-DNA region from pCB301-GFP-HYG into the genome is associated with increased RNA levels of *MAT1-1-1 *and *PPG1 *regardless of the presence or absence of *hph *expression, it was thought that the *Agrobacterium*-mediated transformation process itself could be affecting the expression levels of *MAT1-1-1 *and *PPG1*. To further delineate the effects of *Agrobacterium*-mediated vector integration on RNA levels of *MAT1-1-1*, *PPG1 *and *BEM1*, two additional strains were generated in the G217B background: G217B-blast1 and G217B-blast4. These were generated by random integration of the T-DNA region from a different vector, pCB301-BLAST, into the strain G217B by *Agrobacterium*-mediated transformation. RNA levels of *MAT1-1-1 *and *PPG1 *were elevated in G217B-blast1 and 4 compared to G217B, but levels were not elevated to those found in UC1 (Figure 4A, B). RNA levels of *BEM1 *were similar between G217B-Blast1 and 4, and G217B (Figure 4C). These results indicate that increased *MAT1-1-1 *and *PPG1 *RNA levels in UC1 and UC26 may be partially due to the *Agrobacterium*-mediated transformation process, but again, these increases alone are not sufficient to induce cleistothecia production in the G217-blast strains.

### Overexpression of *MAT1-1-1 *and *BEM1 *in G217B

**Figure 5:**
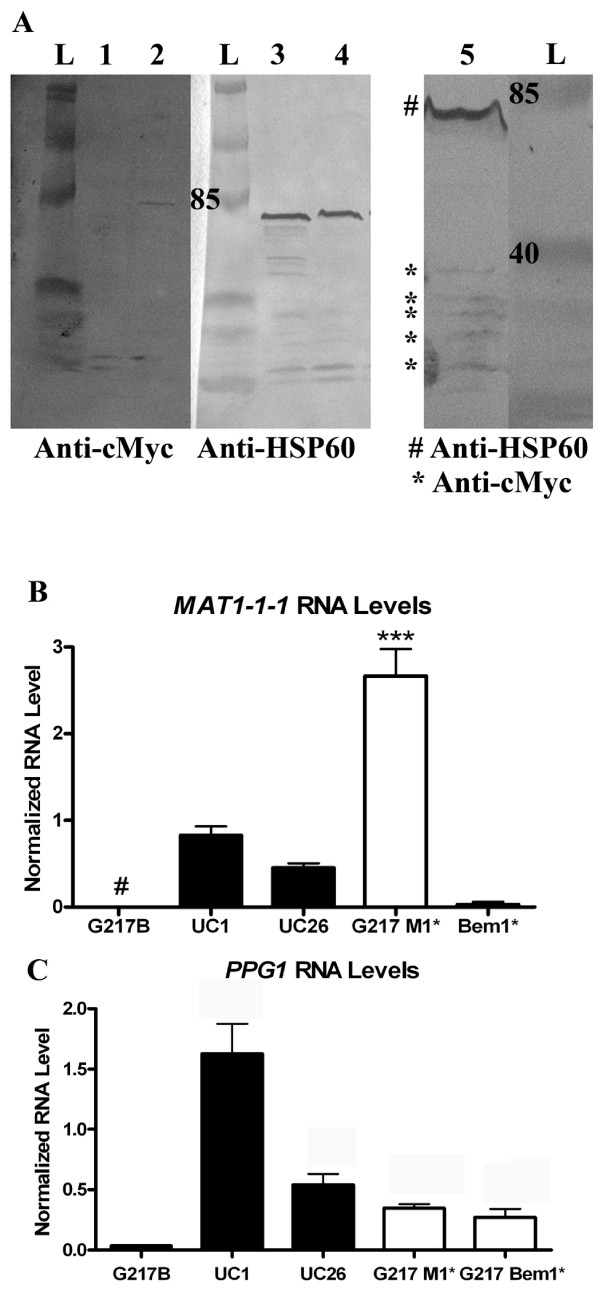
**Overexpression of *MAT1-1-1 *and *BEM1 *in G217B**. A: Detection of c-myc tagged recombinant fusion protein using anti-c-myc antisera on a Western blot of homogenates of *H. capsulatum *strains overexpressing Bem1 (lane 2), Mat1-1-1 (lane 5) or a control strain (lane 1). Detection of HSP60 as a loading control is shown on a duplicate blot in lane 3 and lane 4. B: RNA levels of *MAT1-1-1 *in mycelial phase G217B (n = 4), UC1 (n = 6), and UC26 (n = 4) compared to levels in strains overexpressing *MAT1-1-1 *and *BEM1 *in the G217B background (n = 3) # = below level of detection. **C**: RNA levels of *PPG1 *in mycelial phase G217B (n = 4), UC1 (n = 7), and UC26 (n = 4) compared to levels in strains overexpressing *MAT1-1-1 *and *BEM1 *in the G217B background (n = 3). *** = p ≤ 0.001.

Since strains that are capable of cleistothecia formation exhibited higher RNA levels of *MAT1-1-1*, it was thought that increased expression of this gene could be contributing to cleistothecia production. To determine the effects of increased levels of *MAT1-1-1 *expression on cleistothecia formation, the gene was overexpressed in G217B using the vector pSK-TEL-Kan-Hyg. *BEM1 *was similarly overexpressed in G217B to further assess its role in cleistothecia formation. An irrelevant protein, Kusabira Orange, was expressed in UH3 to provide a hygromycin-resistant mating partner. Proteins of the appropriate size were visible by Western blot of protein extracted from strains overexpressing Bem1 or Mat1-1-1, and then probed with anti-c-Myc antibody (Figure 5A). A UH3-Kusabira Orange strain was crossed with G217B-Bem1* and G217B-Mat1* strains on A-YEM agarose containing hygromycin. No cleistothecia were observed after several months; however, the strains grew slowly on A-YEM with the addition of hygromycin. Predictably, *MAT1-1-1 *RNA levels were increased in the strain overexpressing *Mat1-1-1 *(Figure 5B). RNA levels of *PPG1 *in this strain were also increased compared to levels in G217B, but not to the levels observed in UC1 (Figure 5C). RNA levels of *MAT1-1-1 *were barely detectable in the strain overexpressing Bem1 (Figure 5B), but RNA levels of *PPG1 *in this strain were elevated compared to levels in G217B (Figure 5C). These results indicated that increases in Mat1-1-1 or Bem1 alone are not sufficient to induce cleistothecia production; however, the hygromycin present in the media may have inhibited cleistothecia production by inhibiting the growth of the organisms.

### UC1 as a tool to study cleistothecia formation

**Figure 6:**
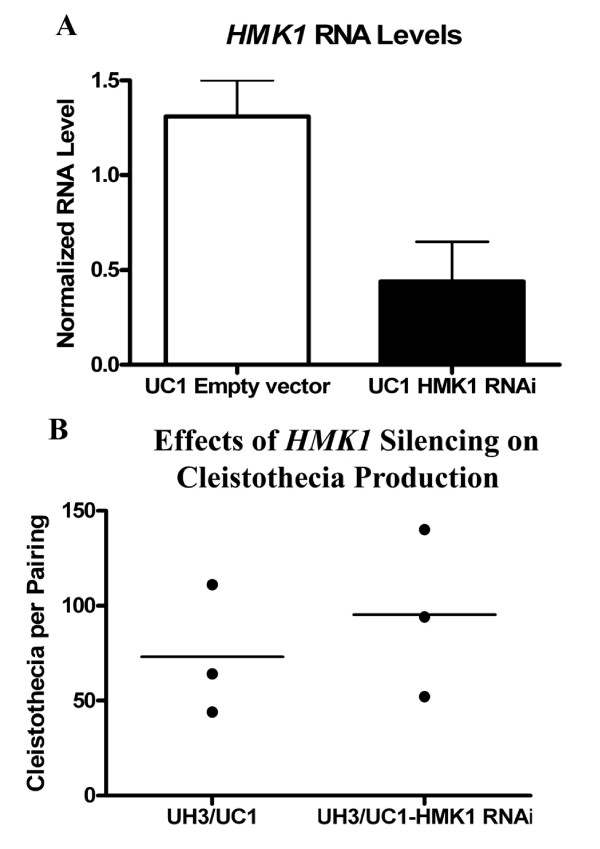
**Effects of silencing HMK1 on cleistothecia formation**. A: *HMK1 *RNA levels found in yeast phase of the silenced strain (UC1-*HMK1*-RNAi) compared to those found in the empty vector control strain by qRT-PCR. Values represent averages and standard error of triplicate samples. B: Number of cleistothecia counted from three pairings of UC1 + UH3, or UC1 with *HMK1 *silenced + UH3.

Although the precise mechanisms by which UC1 gained the ability to form empty cleistothecia remained unclear, the strain provides an opportunity to study cleistothecia production in *H. capsulatum*. Since the pheromone response MAP kinase pathway plays a central role in the mating response of *S. cerevisiae *[12,13], it was predicted to play a similar role in the mating response of *H. capsulatum*. *HMK1*, a putative *FUS3/KSS1 *homolog, was silenced in UC1 to determine the role of the pheromone response pathway in cleistothecia formation of this strain. *HMK1 *RNA levels were reduced to 25% of the levels found in a control strain (Figure 6A). Silencing *HMK1 *had no effect on cleistothecia production when UC1 was paired with UH3 (Figure 6B). This indicates that either the pheromone response pathway is not involved in formation of cleistothecia, or that low levels of *HMK1 *are still sufficient to support cleistothecia formation. Alternatively, the mechanisms that restored cleistothecia production in this strain could be suppressing the effects of silencing *HMK1*.

**Figure 7:**
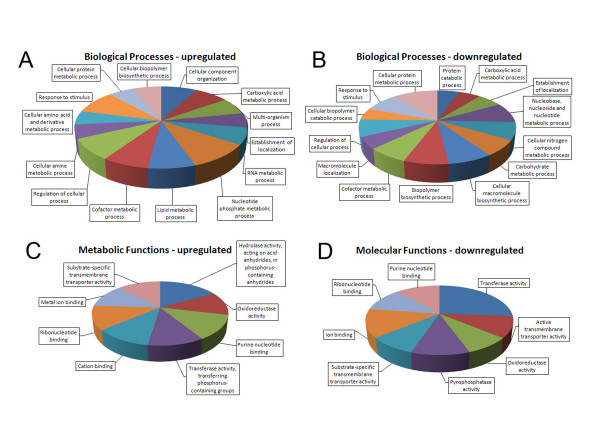
**Microarray analysis of UC26 and G217B gene expression**. Functional annotation of genes up-down regulated greater than 3-fold in UC26 compared to G217B was performed using BLAST2GO Biological processes assigned to upregulated (Panel A) or downregulated genes (Panel B) are shown. Genes with altered gene expression to which molecular function was assigned, are shown in Panel C and D.

To identify additional differences between UC1 and G217B that could play a role in cleistothecia formation, microarray analysis was performed comparing mycelial samples of UC26 and G217B. UC26 was used as the comparator to eliminate the differences attributable to hph activity. Seven hundred and forty one predicted transcripts demonstrated greater than three-fold altered expression in UC26 compared to G217B. Four hundred and thirty four transcripts were upregulated in UC26 compared to G217B while three hundred and nine transcripts were downregulated. Using Blast2Go for blast analysis and assignment of functional annotation and gene ontology, no specific patterns of biological processes could be discerned between up- or downregulated genes (Figure 7). Among genes with assigned molecular functions, genes associated with protein modification or gene regulation, such as transferases and phosphatases, accounted for 37% of downregulated genes in UC26 compared to G217B consistent with the suggestion that no single function results in the acquisition of the ability to form empty cleistothecia.

**Figure 8:**
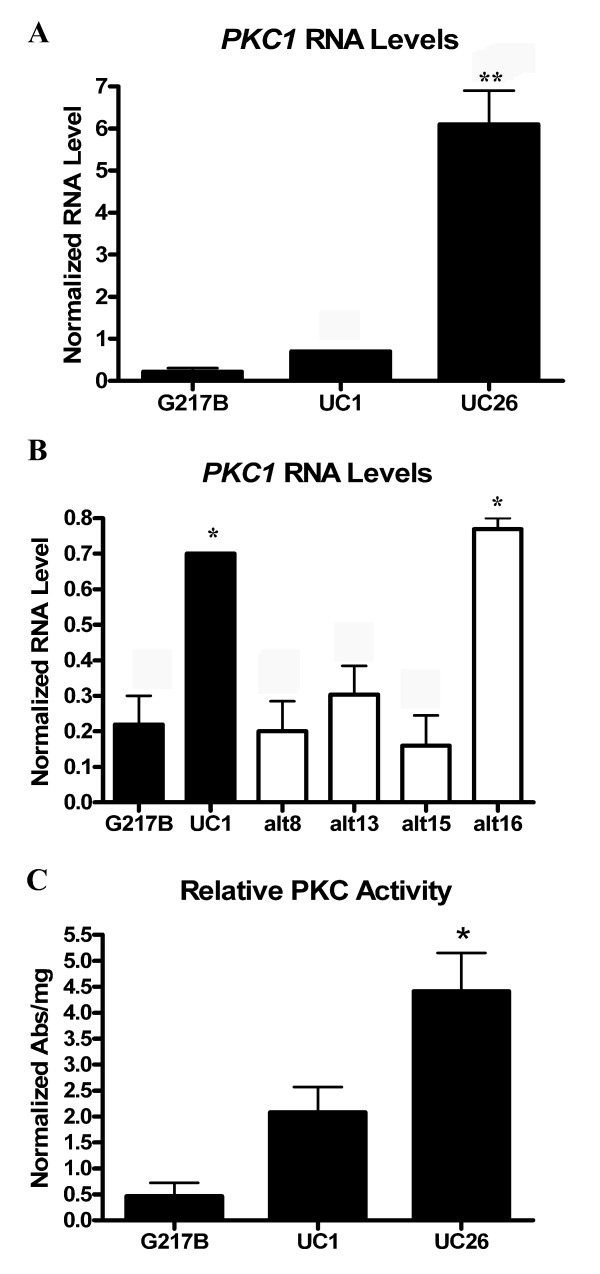
**PKC1 RNA and protein levels in G217B, UC1 and UC26**. A: *PKC1 *RNA levels in mycelial phase G217B, UC1, and UC26, by qRT-PCR. B: *PKC1 *RNA levels in strains with pCB301-HYG-GFP integrated into alternate sites of the genome, compared with *PKC1 *RNA levels in G217B and UC1. C: Pkc1 activity found in activated cell lysates of G217B, UC1, and UC26. All values represent averages and standard error of triplicate samples. * = p ≤ 0.05.

Protein kinase C (*PKC1*) levels were found to be increased 7.16-fold in UC26 compared to G217B (Additional file 1). The elevation on *PKC1 *RNA levels identified by microarray analysis was verified by qRT-PCR in both UC26 and UC1 compared to G217B (Figure 8A). *PKC1 *RNA levels in three of the four strains with T-DNA from the vector pCB301-GFP-HYG integrated at alternate sites were similar to those of G217B (Figure 8B). To determine whether the increased *PKC1 *gene expression resulted in increased protein levels of Pkc1, cytosolic Pkc1 was measured in mycelial cell lysates of G217B, UC1, and UC26. Higher levels of Pkc1 activity were measured in activated cell lysates of UC1 and UC26 compared to G217B (Figure 8C). This indicated that increased levels of Pkc1 in UC1 and UC26 may be contributing to the ability of these organisms to form empty cleistothecia.

**Figure 9:**
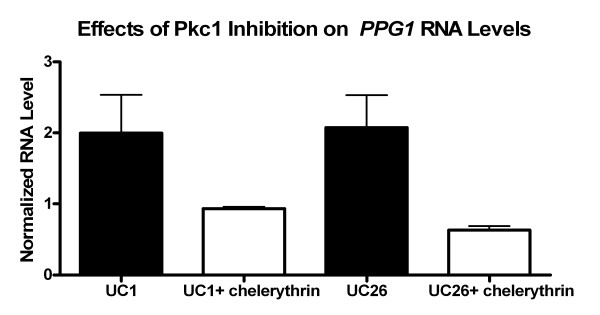
**Effects of PKC inhibitor on pheromone production**. Effects of PKC inhibitor, chelerythrine chloride (25 μM), on *PPG1 *RNA levels in mycelial samples of UC1 and UC26 after 1 hour exposure, compared to UC1 and UC26 exposed to HMM alone. Values represent averages and standard error of triplicate samples.

To further explore the association between increased *PKC1 *levels and cleistothecia formation in *H. capsulatum*, Pkc1 activity of UC1 and UC26 was inhibited by chelerythrine chloride to establish a link between Pkc1 activity and the mating pathway. As previously mentioned, RNA levels of *PPG1 *are elevated in UC1 compared to G217B. Following exposure to 25 μM chelerythrine chloride, *PPG1 *RNA levels decreased in both UC1 and UC26 (Figure 9). These results indicate a link between Pkc1 activity and pheromone production in UC1 and UC26.

## Discussion

Loss of mating ability with continuous culture is not a phenomenon limited to *H. capsulatum*. Strains of *Blastomyces dermatitidis *[25] and *C. neoformans *[26] are also reported to lose mating competency with continuous culture. In one study, mating ability of *C. neoformans *decreased 67% after 600 mitotic generations [26]. Loss of mating ability in cultured fungal organisms may be due to accumulation of mutations in genes that either regulate or are required for mating. The rate of spontaneous mutation has been correlated with loss of mating ability in *C. neoformans *[26]. It has been hypothesized that defects in the *A. fumigatus MAT1-2-1 *regulatory region have led to defects in mating ability of the organism[27], however *A. fumigatus *has recently been shown to be mating competent under certain conditions [28]. The fact that UC1 gained the ability to form empty cleistothecia after a single integrative transformation event indicates that this is an unlikely explanation; however, mutation rates of genes involved in mating have not been analyzed as *H. capsulatum *strains are cultured. This study also did not address the possibility that UC1 gained the ability to form empty cleistothecia due to unidentified genomic rearrangement resulting from the transformation process.

Alternative explanations for loss of mating ability in *H. capsulatum *strains are suggested by the microarray study comparing UC26 and G217B. One possibility is that epigenetic effects play a role in the loss of mating ability demonstrated by *H. capsulatum *strains over time. *C. albicans *white cells switch to the mating-competent opaque form at a higher frequency after being exposed to trichostatin A (TSA), a histone deacetylase inhibitor [29]. Pre-exposure of G217B to TSA for 24 hours does not induce mating ability (data not shown). The Ku proteins, involved in telomeric silencing [30], have also been demonstrated to bind to sites of internal loci and facilitate silencing [31]. *KU80 *RNA levels were found to be decreased 3-fold in UC26 compared to G217B by microarray (Additional file 2). This raises the possibility that the G217B strain may contain genes involved in mating or regulation of mating that have been silenced; however, further verification and studies are required in this area.

Another possibility, suggested by pigmentation observed in the strains studied and supported by the microarray study, is that cAMP levels affect mating competency. The UC1 strain appears more pigmented on HMM plates at room temperature than the G217B strain (data not shown). It has previously been reported that *H. capsulatum *strains lose pigmentation in addition to losing mating ability in culture, and the loss of pigmentation can be used to infer loss of mating competency, through unknown mechanisms [7]. Two putative tyrosinase genes were upregulated in UC26 compared to G217B by microarray (Additional file 1). This may indicate a link between cAMP levels and mating competency. cAMP levels have been shown to regulate melanin production in fungi such as *C. neoformans*, where high cAMP levels stimulate melanin production [32], and *Ustilago hordei*, where high cAMP levels inhibit melanin production [33]. High cAMP levels lead to the activation of PKA [11]. This pathway has been implicated in control of mating in *S. cerevesiae*, where increased PKA activity inhibits sporulation, [34] and impaired PKA activity leads to sporulation even under nutrient-rich conditions that would normally inhibit sporulation [35].

Further comparison of UC1 and G217B, as well as further verification of the microarray data presented here may uncover a molecular mechanism that explains the loss of mating ability in *H. capsulatum*. Additionally, the strain UC1 can be used to study cleistothecia formation in *H. capsulatum*. The cleistothecia formed by the pairing of UC1 and UH3 appear empty. We were unable to detect the presence of asci or ascospores inside the cleistothecia, indicating that the mating process was arrested at some point. The strain UC1 is, therefore, unable to complete the mating process in spite of its ability to form cleistothecia. UC1 does not, however, lose the ability to form empty cleistothecia over time in culture, making it a unique strain that is well suited for studying the molecular and morphological stages of cleistothecia formation.

At this time, it is unclear whether or not hyphal fusion can occur between UC1 and UH3. It is thought that hyphal fusion precedes cleistothecia formation during normal mating in *H. capsulatum *[1], but hyphal fusion may or may not be required for the formation of coiling and branching peridial hyphae comprising the outer structure of the cleistothecia. It is, therefore, unknown at what point the mating process is arrested during the UC1/UH3 cross. The property of the strain UC1 to form empty cleistothecia when crossed with a freshly isolated *MAT1-2 *strain affords the opportunity to dissect the relationship between hyphal fusion and the formation of the outer cleistothecia structure, as well as the contribution of each strain to the mating structure. Although UC1 contains a functional GFP gene, its expression is under control of the calcium binding protein gene promoter and is therefore limited to expression in yeast phase organisms. Because mating occurs in the mycelial phase, an additional derivative of UC1 expressing a fluorescent marker in the mycelial phase would need to be generated to answer these questions.

There is no clear pattern of pheromone and pheromone receptor expression under standard growth conditions in the *H. capsulatum *strains studied here. In *S. cerevisiae*, MAT**a **strains secrete a pheromone and express the alpha pheromone receptor *STE2*, while MATalpha strains secrete alpha pheromone and express the a pheromone receptor *STE3 *[36]. There are also, however, examples of fungi such as *Neurospora crassa *in which both pheromone receptors are constitutively expressed [37]. In the current study, *STE2 *RNA levels were elevated in the established laboratory strain G217B, while *STE3 *levels were undetectable. The fact that *STE2 *but not *STE3 *is detected in G217B would indicate that organisms of *MAT1-1 *mating type are responsive to alpha pheromone. This would confirm previous studies, which showed *MAT1-1-1 *RNA levels in a clinical *H. capsulatum *strain were responsive to an extract enriched for alpha pheromone [2]. If *MAT1-1 *strains respond to alpha pheromone, they would be expected to produce a pheromone. However UC1, the strain capable of empty cleistothecia formation, produces elevated RNA levels of alpha pheromone. The a pheromone gene has yet to be identified in *H. capsulatum*.

RNA levels of both *STE2 *and *STE3 *are also detectable in UC1. In *A. fumigatus*, strains of both mating types also express alpha pheromone and both pheromone receptors under a variety of conditions [38]. It may be that the correct combination of stimulation and growth conditions is required in these organisms to observe only one pheromone and pheromone receptor expressed exclusively in each organism of opposite mating type. Incorrect expression of pheromone receptors has been shown to affect mating ability in *S. cerevisiae*, as MATa cells also expressing a pheromone receptor do not undergo G1 arrest when exposed to alpha pheromone [39]. As pheromone receptor expression patterns differ between G217B and UC1, this could play a role in UC1's ability to form empty cleistothecia, or in UC1's inability to form ascospores.

Both RNA and cytosolic protein levels of Pkc1 are increased in UC1 and UC26 compared to G217B. Pkc1 has not previously been directly connected to the pheromone response pathway in any fungal organism. *PKC1 *is connected to the pheromone response pathway through crosstalk in *S. cerevisiae*, where the cell wall integrity pathway and the pheromone response pathway are both activated by pheromone [18,40]. *PKC1 *is required for the crosstalk between the pheromone response pathway and the cell integrity pathway in *S. cerevisiae*, which is, in turn, required for mating [40]. Our studies showed that silencing *HMK1*, the predicted MAP kinase involved in the pheromone response pathway, had no effect on cleistothecia production in UC1. It is possible that the pheromone response MAP kinase pathway plays a minimal role in cleistothecia production of *H. capsulatum*. The pheromone response pathway may be playing a greater role in other aspects of the mating process, such as ascospore formation. Since the UC1 strain forms empty cleistothecia and the reasons for the lack of ascospore formation are unknown, it would be difficult to define the role of the pheromone response pathway in any aspect of mating besides cleistothecia production using this strain. Future studies will, however, be able to address the role of the cell wall integrity pathway in cleistothecia production using the UC1 strain.

## Conclusions

In conclusion, we generated a laboratory strain of *H. capsulatum*, UC1, by insertional mutagenesis of a mating incompetent strain that was subsequently able to form empty cleistothecia with a recent clinical isolate. We determined that RNA levels of genes involved in the mating process are increased in UC1, and that the T-DNA insertion site plays a role in the strain's ability to form empty cleistothecia. Using UC1 as a tool to study cleistothecia production, we determined that *PKC1 *RNA levels are increased in UC1 and UC26. We established a link between Pkc1 activity and pheromone production by showing that a PKC inhibitor decreases RNA levels of *PPG1 *in UC1 and UC26. Pkc1 is tenuously linked with mating ability in other fungi, and further studies in *H. capsulatum *are required to provide evidence of a direct link between mating ability and Pkc1 activity. Future studies in cleistothecia production of *H. capsulatum *may provide a means to prevent or reverse the loss of mating ability as this organism is cultured in the laboratory.

## Methods

### Strains and growth conditions

*H. capsulatum *strain G217B (ATCC 26032) was a kind gift from George Deepe, University of Cincinnati, Cincinnati, OH. Generation of UC1, a GFP-expressing derivative of G217B, has previously been described (40). UH3 was a clinical isolate. UH1 was a clinical isolate obtained from a transplant patient with disseminated histoplasmosis, and VA1 was a clinical isolate obtained from a human immunodeficiency virus/AIDS patient with disseminated histoplasmosis. Yeast phase organisms were maintained on *Histoplasma *macrophage medium (HMM) plates at 37°C under 5% CO_2 _in a humidified incubator. Mycelial phase cultures were generated by streaking yeast phase organisms growing at 37°C onto a nylon filter (Millipore) placed on an HMM plate, and were grown at 25°C. Liquid cultures grown in HMM were started from organisms growing on HMM plates at 37°C, and then grown at 37°C in an orbital shaker. Plates and media were supplemented with 200 μg/mL hygromycin or 100 μg/mL blasticidin S when appropriate.

### Strain generation

#### UC26

*Histoplasma capsulatum *strain UC26 was generated from strain UC1 by liberation of the *Aspergillus nidulans gpd *promoter-*E. coli hph*-*A. nidulans trpC *terminator sequence fragment by Cre-mediated recombination. Briefly, a general purpose *H. capsulatum *shuttle vector pSK-Tel-Kan-Blast was constructed by fusion of (i) the backbone of pSKII+ containing the origin of replication and multiple cloning site with (ii) a fragment from pCR83 containing *H. capsulatum *telomere sequence repeats flanking the kanamycin resistance cassette and (iii) a fragment containing the *A. terreus *blasticidin deaminase gene *bsd *under control of the *A. nidulans gpd *promoter and flanked by the *A. nidulans trpC *terminator. Fragments with compatible end sequences were generated by standard PCR amplification. A similar vector pSK-Tel-Kan-Hyg was generated using a hygromycin resistance cassette comprising the *A. nidulans gpd *promoter-*E. coli hph*-*A. nidulans trpC *terminator sequence in place of the blasticidin resistance cassette. The *H. capsulatum cbp *promoter was amplified using pCR83 as template and fused to the Cre cDNA obtained from the plasmid pSMP8-Cre (a gift from Dr. Tom Clemens) and the *H. capsulatum ura5 *terminator sequence. The *cbp *promoter-Cre cDNA-u*ra5 *terminator fragment was ligated into pSK-Tel-Kan-Blast. Ligation junctions and other critical sequence regions were verified by sequencing across the junctions. The resulting plasmid containing the Cre cDNA under control of the *cbp *promoter was linearized and electroporated into *H. capsulatum UC1 *under standard conditions. Transformants were selected on HMM plates containing 100 μg/mL blasticidin S and subsequently serially passaged in liquid HMM media supplemented with 100 μg/mL blasticidin S for 3 passages and placed on HMM-blasticidin plates. Individual colonies were replica plated to HMM plates supplemented with 200 μg/mL hygromycin. The loss of the *hph *gene was verified by PCR using *hph *specific primers in clones unable to grow in the presence of hygromycin. One such clone was selected and cured of the presence of the pSK-Tel-Kan-Blast-Cre plasmid by repeat passage in media in the absence of blasticidin selection. Loss of the plasmid was demonstrated phenotypically by the development of blasticidin S sensitivity and verified by the failure to amplify the bsd gene sequence. This clone was designated *H. capsulatum *UC 26.

#### ALT8, ALT13, ALT15, ALT16

The ALT strains were generated by *Agro bacterium*-mediated transformation of T-DNA from the vector pCB301-GFP-HYG into the G217B strain as previously described [21,23,24]. The site of integration of each strain was identified by TAIL-PCR as previously described, and verified to each be unique and distinct from that of UC1 [40].

#### ALT-Cre1, ALT-Cre2

The ALT-Cre strains was generated by excision of the *A. nidulans gpd *promoter-*E. coli hph*-*A. nidulans trpC *terminator sequence fragment from ALT-16 by Cre-mediated recombination as described above.

#### UC1-*HMK1*-RNAi

An *Agrobacterium *binary vector for RNAi mediated silencing pCB301-Blast-186 was generated by the fusion of the *A. nidulans gpd *promoter-*bsd *gene-*A. nidulans trpC *terminator cassette described above with an *Eco*RI- *Bsp*DI fragment liberated from pCR186 (obtained from Drs. William Goldman and Chad Rappleye) containing the *H. capsulatum *H2B promoter sequences driving expression of a chimeric hairpin RNAi construct containing a portion of the GFP gene and a gene of interest flanked by the *H. capsulatum catB *terminator sequence. T-DNA from the vector pCB301-Blast-186 was transformed into UC1 as described previously [23,24]. For the control strain, the hairpin construct contained sequence only for GFP.

#### G217B-Blast1, G217B-Blast4, UH3-Blast

The Blast strains were generated by *Agrobacterium*-mediated transformation of T-DNA from the vector pCB301-Blast-186 described above, into G217B or UH3, as described previously [23,24].

#### G217B-Mat1* and G217B-Bem1*

To facilitate the express of recombinant proteins in *H. capsulatum*, the H2B promoter was amplified generating a *Apa*I-H2B-*Asc*I fragment which was ligated to a synthetic oligonucleotide comprising an *Asc*I site, an irrelevant stuffer sequence, a *Sbf*I site and sequence encoding the cMyc epitope and in-frame stop codon. This was ligated to the *H. capsulatum catB *terminator sequence amplified with a downstream *Xba*I site. The fused fragment was ligated into the polylinker sequence of pSK-Tel-Kan-Hyg between the *Apa*I and *Spe*I sites to generate the overexpression vector pSK-Tel-Kan-Hyg-H2B-cMyc-catBterm. cDNA fragments encoding HcBem1 or Mat1-1-1were ligated between the *Asc*I and *Sbf*I sites. Following linearization with *Pme*I, the telomeric expression vectors were electroporated into *H. capsulatum *G217B under standard conditions. Hygromycin resistant transformants expressed cMyc-tagged Bem1 or cMyc-tagged Mat1-1-1 fusion proteins.

### Observation of cleistothecia-like structure production

Organisms were streaked onto a nylon filter (Millipore), placed on an HMM plate, and grown at 25°C until mycelial growth was observed. A small amount of each mycelia mat was transferred to an Alphacel Yeast Extract medium (A-YEM) agarose plate and mixed with organisms of the opposite mating type. Plates were sealed with Petri-seal tape, and organisms were grown for one month at 25°C in the dark. After this time, cleistothecia were visible to the naked eye. Cleistothecia were either counted with the aid of a dissecting microscope, or lifted from the plate using clear Petri-seal tape. Organisms were fixed with lactophenol mounting media and examined by light microscopy using a Nikon E600 microscope and a SPOT RT slider camera, or confocal microscopy using a Nikon PCM2000 laser scanning confocal microscope and SimplePCI^© ^software.

### Scanning electron microscopy

UH3 and UC1 were grown together on A-YEM as described above until cleistothecia were visible (about one month). Organisms were lifted from the plated using Petri-seal tape and fixed using low concentration Karnovsky's Fixative from Electron Microscopy Sciences (2% paraformaldehyde, 2.5% glutaraldehyde, and 0.1 M Sodium Phosphate buffer). Selected cleistothecia were dissected using syringe needles. SEM imaging was performed on samples containing dissected and whole cleistothecia by RealView Analytical Laboratory. Samples were rinsed with PBS solution, dehydrated through a series of 50%, 70%, 85%, 95%, 100%, 100% ethanol aqueous solutions, air dried, and then coated with a ~100 nm layer of carbon. The coated samples were viewed under an FEI/Philips XL30 ESEM, and digital images were taken.

### Quantitative real-time RT-PCR (qRT-PCR)

Strains were grown in mycelial or yeast phase as described above. Experimental samples were collected in triplicate unless otherwise noted. RNA was extracted using TRIzol^® ^reagent (Invitrogen) according to manufacturer's instructions. cDNA synthesis was performed using random primers and Superscript II reverse transcriptase (Invitrogen). qRT-PCR was performed in triplicate for each sample using the Applied Biosystems 7500 Real-time PCR system (Applied Biosystems). SYBR green PCR mastermix (Applied Biosystems) was used for detection. Primers used are listed in Table 2. One primer of each qRT-PCR primer pair was designed to span an intron, and each primer pair was tested for the inability to amplify genomic DNA. RNA levels were obtained using a standard curve generated from a pool of all samples tested. Normalized RNA levels were calculated using the standard curve method for relative quantification, using *GAPDH *RNA levels as an endogenous reference. Outliers were removed using Grubb's test for detecting outliers, and normalized RNA levels were compared using the Tukey-Kramer Multiple Comparisons Test (GraphPad, Instat).

**Table 2:** qRT-PCR primers

***GAPDH***	S: ATTGGGCGTATTGTCTTCC AS: TTGAGCATGTAGGCAGCATA
***MAT1-1-1***	S: TTCGTTCATAGCCTTCAGAAGCTTC AS: GGCCAGCATGACTGTCACGAAT
***PPG1***	S: CTGTGTGGGCAGTAGCAATTCC AS: CAACGTTTCGCGACGAATTCA
***STE2***	S: TGCTCTCGTTACTCGCAGAC AS: ATTGGATTATTGAGAAAATGGCTGGAATC
***STE3***	S: ACAATCGGTATATAACCAATACACAGTAG AS: GTTGTCCAGCACCGTCGATA
***BEM1***	S: TGGAAGAAGATGACGGCGGAAT AS: TGTGGCTTTGTTGTAGGTGAGGG
***HMK1***	S: CGTGGCAGCACAGACAATGC AS: GGCGGATTTGCAAGGACGT
***PKC1***	S: CCGAAAGTCGTCACCAAGTG AS: CATGTAAGACTGCATTCTGAGC

### Western Blot

An amount of organism equivalent to 10 μL was taken from an HMM plate kept at 37°C. 30 μL of Laemelli sample buffer was added and samples were boiled for 15 min. Samples were electrophoresed by SDS-PAGE using a precast 8-16% tris-glycine gel (Invitrogen) and transferred to a nitrocellulose membrane. Membranes were either incubated at room temperature for 2 hours with a 1:1000 dilution of anti-Myc alkaline phosphatase tagged antibody (Invitrogen), or with a 1:5000 dilution of rabbit anti-HSP60 antibody (a kind gift from Francisco Gomez, University of Cincinnati, Cincinnati, OH) as a loading control. Anti-HSP60 antibodies were secondarily tagged with a 1:1000 dilution of peroxidase labelled goat-anti-rabbit antibody (Kirkegaard and Perry Laboratories). Phosphatase labelled antibodies were developed using BCIP/NBT phosphatase substrate (Kirkegaard and Perry Laboratories), and peroxidase labelled antibodies were developed using TMB One Component, HRP membrane substrate (BioFx Laboratories). Developed membranes were imaged using a FOTO/Analyst^® ^FX system from Fotodyne^®^, Inc.

### Microarray

Microarray analysis was performed in conjunction with the University of Cincinnati Genomics and Microarray laboratory. Three samples of G217B and UC26 were grown at 25°C on nylon membranes placed on HMM plates as described above. RNA was extracted using TRIzol^® ^reagent (Invitrogen) according to manufacturer's instructions. Cy-3 and Cy-5 labelled cDNA from UC26 and G217B was hybridized to a slide containing 70-mer oligonucleotides representing each putative open reading frame in the *H. capsulatum *genome (Washington University Genome Sequencing Center, Washington University, St. Louis, MO). Dye swaps were also performed. Slides were imaged using a GenePixPro 4000 scanner (Axon Instruments), using Axon GenePix^® ^Pro version 5.0 software. Cy3 and Cy5 intensities were normalized by subtracting local background intensities from the median intensity of each channel. Statistical analysis was performed by the University of Cincinnati Bioinformatics F&S Core of the Center for Environmental Genetics, as previously described [41]. Functional analysis was performed using BLAST2GO http://www.blast2go.org which performed Blast analysis against public databases, mapping against GO resources to fetch functional data and annotation to generate functional assignments. Raw microarray data has been submitted to the Gene Expression Omnibus (GEO) repository under the accession number GSE19762.

### Protein kinase C assay

UC1, UC26, and G217B were grown on nylon filters at 25°C as described above. After growth was observed, cells were lysed, and the non-radioactive protein kinase assay kit (Calbiochem) was used to activate PKC in the cell lysates and measure PKC activity according to the manufacturer's instructions. The experiment was performed in triplicate. Outliers were removed using Grubb's test. Results were compared using the Tukey-Kramer Multiple Comparisons Test (GraphPad, Instat).

### Protein kinase C inhibition study

UC1 and UC26 were grown on nylon filters at 25°C as described above. After growth was observed (about 1 week), the membrane was placed fungus side down into a petri dish containing HMM media, or HMM media supplemented with 100 μM chelerythrine chloride (Sigma) from a 5 mg/mL stock solution dissolved in water. The experiment was performed in triplicate for each strain. After one hour, RNA was extracted, and qRT-PCR was performed as described above. *GAPDH *RNA levels were similar to those measured in previous experiments, indicating that the cells were not dying due to the PKC inhibitor.

## Authors' contributions

MCL performed the molecular genetics, protein, and mating studies, and drafted the document. AGS generated strains and molecular reagents, directed design and coordination of the studies, and helped draft the document. Both authors have read and approved the final manuscript.

## Supplementary Material

Additional file 1 **Genes upregulated in UC26 vs G217B**. This file contains a listing of all genes upregulated 3 fold or more in *H. capsulatum *strain UC26 compared to G217B. The data includes the *H. capsulatum *gene name, the gene annotation and the fold change. Click here for file

Additional file 2 **Genes downregulated in UC26 vs G217B**. This file contains a listing of all genes downregulated 3 fold or more in *H. capsulatum *strain UC26 compared to G217B. The data includes the H. capsulatum gene name, the gene annotation and the fold change. Click here for file
