# Supplementary material for: Insertional mutagenesis enables cleistothecial formation in a non-mating strain of Histoplasma capsulatum
Source: BMC Microbiol. 2010 Feb 16;10:49. doi: 10.1186/1471-2180-10-49 (PMC2834667; doi:10.1186/1471-2180-10-49)
Supplement: Additional file 1 — Genes upregulated in UC26 vs G217B. This file contains a listing of all genes upregulated 3 fold or more in H. capsulatum strain UC26 compared to G217B. The data includes the H. capsulatum gene name, the gene annotation and the fold change. [file 1471-2180-10-49-S1.DOC]

### Additional file 1 – Genes upregulated in UC26 vs G217B

| ***H. capsulatum*** **gene names[42]:** | **Gene annotation[42]:** | **Fold change** |
| --- | --- | --- |
| HISTO_ZE.Contig158.Fgenesh_Aspergillus.56.final_new | YKT6 SGDID:S000001679 Vesicle membrane protein (v-SNARE | 60.32 |
| HISTO_JG.Contig207.eannot.1420.final_new | NTF2 SGDID:S000000811 Nuclear envelope protein | 23.19 |
| HISTO_ZT.Contig1129.eannot.1089.final_new | SUR7 SGDID:S000004516Integral membrane protein localized to cortical patch structures | 20.35 |
| HISTO_ER.Contig17.Fgenesh_histo.70.final_new | Aspergillus fumigatus: HET domain protein, putative | 19.88 |
| HISTO_ZU.Contig65.fgenesh_plus.93.final_new | SER3 SGDID:S000000883 3-phosphoglycerate dehydrogenase | 11.75 |
| HISTO_ZY.Contig518.Fgenesh_histo.31.final_new | RBE1 CGDID:CAL0003775 Putative cell wall protein | 11.52 |
| HISTO_ZZ.Contig127a.Fgenesh_histo.76.final_new | Aspergillus fumigatus: C6 transcription factor, putative | 11.51 |
| HISTO_ZL.Contig658.eannot.1289.final_new | RIM101 SGDID:S000001019 Transcriptional repressor, pH | 11.37 |
| HISTO_ZE.Contig158.eannot.1424.final_new | Aspergillus fumigatus: FAD monooxygenase, putative | 11.16 |
| HISTO_ZU.Contig65.eannot.2055.final_new | HTA2 SGDID:S000000099 Histone H2A | 10.69 |
| HISTO_GX.Contig297.genewise.55.final_new | MSW1 CGDID:CAL0000390 mitochondrial tryptophanyl-tRNA synthetase | 10.64 |
| HISTO_GY.Contig460.Fgenesh_Aspergillus.137.final_new | GCD7 SGDID:S000004282  Beta subunit of the translation initiation factor eIF2B | 10.21 |
| HISTO_GL.Contig296.Fgenesh_histo.88.final_new | PCK1 SGDID:S000001805 Phosphoenolpyruvate carboxykinase | 9.93 |
| HISTO_KF.Contig601.Fgenesh_Aspergillus.70.final_new | FAT1 SGDID:S000000245  Fatty acid transporter and very long-chain fatty acyl-CoA synthetase | 9.73 |
| HISTO_DY.Contig31.fgenesh_plus.50.final_new | Aspergillus fumigatus: alcohol dehydrogenase | 9.51 |
| HISTO_EA.Contig33.eannot.1577.final_new | SUI1 SGDID:S000005188 Translation initiation factor eIF1 | 9.5 |
| HISTO_DU.Contig190.Fgenesh_histo.69.final_new | HOL1 SGDID:S000005338 Putative ion transporter | 9.13 |
| HISTO_ZL.Contig1131-snap.235.final_new | DML1 SGDID:S000004824 Essential protein involved in mtDNA inheritance | 8.45 |
| HISTO_JG.Contig207.eannot.1427.final_new | TID3 SGDID:S000001406 Component of the kinetochore-associated Ndc80 complex | 8.37 |
| HISTO_KF.Contig601.Fgenesh_histo.18.final_new | Neurospora crassa: tyrosinase precursor (Monophenol monooxygenase) | 8.03 |
| HISTO_HS.Contig68.Fgenesh_Aspergillus.86.final_new | MAL31 SGDID:S000000502 Maltose permease | 7.55 |
| HISTO_FE.Contig19.eannot.1765.final_new | XOG1 CGDID:CAL0006153 Exo-1,3-beta-glucanase, major exoglucanase | 7.48 |
| HISTO_LG.Contig392.eannot.1721.final_new | NOP10 SGDID:S000007455 small nucleolar ribonucleoprotein particles | 7.47 |
| HISTO_GL.Contig296.Fgenesh_histo.128.final_new | Aspergillus fumigatus: bZIP transcription factor (AP-1), putative | 7.32 |
| HISTO_DY.Contig31.Fgenesh_histo.61.final_new | GIN4 SGDID:S000002915Protein kinase involved in bud growth and assembly of the septin ring | 7.18 |
| HISTO_LF.Contig359.Fgenesh_histo.161.final_new | PKC1 SGDID:S000000201 Protein Kinase C | 7.16 |
| HISTO_GX.Contig297.eannot.1512.final_new | Aspergillus fumigatus: alpha/beta hydrolase, putative | 7.08 |
| HISTO_FE.Contig19.eannot.1802.final_new | Neurospora crassa: related to ubiquitin-protein ligase HUL4 | 7.02 |
| HISTO_ZL.Contig1161c.eannot.1522.final_new | Gibberella zeae: TYR1, putative tyrosinase | 6.92 |
| HISTO_DM.Contig933.Fgenesh_histo.171.final_new | MSH6 SGDID:S000002504, Protein required for mismatch repair | 6.9 |
| HISTO_ZL.Contig1131-snap.231.final_new | DEG1 SGDID:S000001895 Non-essential tRNA pseudouridine synthase | 6.71 |
| HISTO_KF.Contig470.Fgenesh_histo.2.final_new | Coccidioides posadasii: chitinase 3 | 6.53 |
| HISTO_HS.Contig68.eannot.2162.final_new | PHO84 CGDID:CAL0002095 Protein similar to high-affinity phosphate transporters | 6.49 |
| HISTO_ZT.Contig174.eannot.1420.final_new | CDC123 SGDID:S000004205 Protein involved in nutritional control of the cell cycle | 6.46 |
| HISTO_ER.Contig17.Fgenesh_histo.25.final_new | URA5 SGDID:S000004574 | 6.43 |
| HISTO_GL.Contig233-snap.6.final_new | LSP1 SGDID:S000005925 inhibitor of protein kinases Pkh1p and Pkh2p | 6.3 |
| HISTO_LG.Contig392.Fgenesh_histo.47.final_new | DIP5 SGDID:S000006186 Dicarboxylic amino acid permease | 6.25 |
| HISTO_FJ.Contig643.fgenesh_plus.4.final_new | Aspergillus fumigatus: oxidoreductase, putative | 6.24 |
| HISTO_ZU.Contig65.Fgenesh_Aspergillus.286.final_new | CTR9 SGDID:S000005505 Component of the Paf1p complex; | 5.94 |
| HISTO_LF.Contig359.eannot.1931.final_new | YRB1 SGDID:S000002409 Ran GTpase binding protein | 5.9 |
| HISTO_FX.Contig167.eannot.1335.final_new | Coccidioides posadasii: opsin 1 | 5.78 |
| HISTO_ZE.Contig149.eannot.1274.final_new | YCS4 SGDID:S000004262 Non-SMC subunit of the condensin complex | 5.76 |
| HISTO_GY.Contig471.eannot.1321.final_new | SSU1 CGDID:CAL0004538 sulfite transport protein | 5.72 |
| HISTO_ZL.Contig1161d.eannot.1494.final_new | SWS2 SGDID:S000005025 Putative mitochondrial small ribosomal subunit | 5.68 |
| HISTO_ZL.Contig1158.Fgenesh_histo.81.final_new | SYP1 SGDID:S000000626 Protein with a potential role in actin cytoskeletal organization | 5.48 |
| HISTO_KF.Contig597.Fgenesh_Aspergillus.79.final_new | FRQ1 SGDID:S000002781 N-myristoylated calcium-binding protein | 5.45 |
| HISTO_LF.Contig359.Fgenesh_histo.7.final_new | RIA1SGDID:S000005107  Cytoplasmic GTPase involved in biogenesis of the 60S ribosome | 5.45 |
| HISTO_ZH.Contig107.genewise.17.final_new | TNA1 SGDID:S000003492 High affinity nicotinic acid plasma membrane permease | 5.31 |
| HISTO_ZL.Contig658.Fgenesh_Neurospora.79.final_new | PTR2 SGDID:S000001801  Integral membrane peptide transporter | 5.23 |
| HISTO_ZT.Contig181.Fgenesh_Aspergillus.165.final_new | HCM1 SGDID:S000000661 Forkhead transcription factor involved in cell cycle specific transcription of SPC110 | 5.22 |
| HISTO_DM.Contig936.eannot.1239.final_new | ARD1 SGDID:S000001055  Subunit of the N-terminal acetyltransferase NatA | 5.14 |
| HISTO_DU.Contig204.fgenesh_plus.3.final_new | VID24 SGDID:S000000309, Peripheral membrane protein located at vacuole import and degradation vesicles | 5.1 |
| HISTO_ZE.Contig158.eannot.1345.final_new | CTT1 SGDID:S000003320 Cytosolic catalase T | 4.96 |
| HISTO_ZL.Contig1161c-snap.24.final_new | TRM10 SGDID:S000005453 tRNA methyltransferase | 4.8 |
| HISTO_ZT.Contig174.eannot.1384.final_new | CTT1 SGDID:S000003320 Cytosolic catalase T | 4.8 |
| HISTO_GL.Contig296.Fgenesh_histo.51.final_new | UPC2 SGDID:S000002621 Sterol regulatory element binding protein | 4.73 |
| HISTO_LG.Contig392.eannot.1712.final_new | SPT8 SGDID:S000004045  Subunit of the SAGA  transcriptional regulatory complex | 4.73 |
| HISTO_ZL.Contig1161e.eannot.1224.final_new | DSE4 SGDID:S000005350 Daughter cell-specific secreted protein with similarity to glucanases | 4.65 |
| HISTO_ZT.Contig1128f-snap.1.final_new | TAD1 SGDID:S000003212 tRNA-specific adenosine deaminase | 4.45 |
| HISTO_ZL.Contig1161e-snap.47.final_new | VPS52 SGDID:S000002892 Component of the GARP | 4.4 |
| HISTO_ZL.Contig1117.eannot.1111.final_new | RFC3 SGDID:S000005234 Subunit of heteropentameric replication factor C | 4.38 |
| HISTO_GY.Contig460.genewise.7.final_new | ATG7 SGDID:S000001214 Autophagy-related protein | 4.37 |
| HISTO_ZY.Contig518.eannot.1172.final_new | RCY1 SGDID:S000003740 F-box protein involved in recycling plasma membrane proteins | 4.37 |
| HISTO_KK.Contig134.eannot.1133.final_new | RNR1 SGDID:S000000872 Ribonucleotide-diphosphate reductase (RNR), large subunit | 4.36 |
| HISTO_ZT.Contig1089.eannot.1542.final_new | ACO1 CGDID:CAL0001406 Protein described as aconitase | 4.3 |
| HISTO_EA.Contig33.genewise.118.final_new | UBX7 SGDID:S000000477 Ubiquitin regulatory X domain-containing protein | 4.25 |
| HISTO_ZT.Contig181.eannot.1695.final_new | POL2 SGDID:S000005206 Catalytic subunit of DNA polymerase epsilon | 4.21 |
| HISTO_LF.Contig359.Fgenesh_histo.163.final_new | HEM1 SGDID:S0000026405 -aminolevulinate synthase | 4.2 |
| HISTO_LAE.Contig1.Fgenesh_Aspergillus.2.final_new | SGS1 SGDID:S000004802 Nucleolar DNA helicase of the RecQ family | 4.18 |
| HISTO_EA.Contig33.eannot.1563.final_new | HHO1 SGDID:S000006048 Histone H1 | 3.98 |
| HISTO_ZT.Contig174.eannot.1401.final_new | ADE4 SGDID:S000004915 Phosphoribosylpyrophosphate amidotransferase | 3.95 |
| HISTO_LG.Contig392-snap.157.final_new | IMH1 SGDID:S000004300 Protein involved in vesicular transport | 3.94 |
| HISTO_GY.Contig460.Fgenesh_histo.160.final_new | KHA1 SGDID:S000003630 Putative K+/H+ antiporter | 3.93 |
| HISTO_ZL.Contig1161e.eannot.1288.final_new | GYP8 SGDID:S000001867 GTPase-activating protein | 3.9 |
| HISTO_ZY.Contig562f.Fgenesh_Aspergillus.10.final_new | DNM1 SGDID:S000003924 Dynamin-related GTPase | 3.9 |
| HISTO_ZL.Contig1131.eannot.2025.final_new | FOL1 SGDID:S000005200 Multifunctional enzyme of the folic acid biosynthesis pathway | 3.89 |
| HISTO_GL.Contig233.Fgenesh_histo.40.final_new | TYS1 SGDID:S000003417 Cytoplasmic tyrosyl-tRNA synthetase | 3.84 |
| HISTO_ZT.Contig1089.genewise.39.final_new | KIP2 SGDID:S000006076 Kinesin-related motor protein | 3.84 |
| HISTO_ZZ.Contig127c.Fgenesh_histo.126.final_new | PMC1 SGDID:S000002974 Vacuolar Ca2+ ATPase involved in depleting cytosol of Ca2+ ions | 3.81 |
| HISTO_GY.Contig460.Fgenesh_Aspergillus.54.final_new | PGS1 SGDID:S000000510 Phosphatidylglycerolphosphate synthase | 3.8 |
| HISTO_GY.Contig460.Fgenesh_histo.226.final_new | RAD5 SGDID:S000004022 Single-stranded DNA-dependent ATPase | 3.8 |
| HISTO_ZZ.Contig2f.eannot.1074.final_new | BNA6 SGDID:S000001943 Quinolinate phosphoribosyl transferase | 3.75 |
| HISTO_FE.Contig19.Fgenesh_Aspergillus.226.final_new | Aspergillus fumigatus: endo-1,3(4)-beta-glucanase, putative | 3.68 |
| HISTO_GL.Contig296.Fgenesh_histo.27.final_new | KRS1 SGDID:S000002444 Lysyl-tRNA synthetase | 3.67 |
| HISTO_LY.Contig16.Fgenesh_histo.9.final_new | HEX1 CGDID:CAL0004108 Beta-N-acetylhexosaminidase (beta-N-acetylglucosaminidase/chitobiase) | 3.66 |
| HISTO_FE.Contig19.Fgenesh_histo.134.final_new | Aspergillus nidulans: HSP90 | 3.64 |
| HISTO_LF.Contig359.eannot.2106.final_new | IMP1 SGDID:S000004758Catalytic subunit of the mitochondrial inner membrane peptidase complex | 3.62 |
| HISTO_EA.Contig33.Fgenesh_histo.140.final_new | FAA2-1 CGDID:CAL0005590 Predicted long chain fatty acid CoA ligase | 3.6 |
| HISTO_FO.Contig19.Fgenesh_histo.2.final_new | FUM1 SGDID:S000006183 Fumarase | 3.57 |
| HISTO_GL.Contig296.eannot.1402.final_new | DIC1 SGDID:S000004340 Mitochondrial dicarboxylate carrier | 3.49 |
| HISTO_ZT.Contig174.genewise.95.final_new | NEO1 SGDID:S000001310 Protein involved in retrograde transport from the Golgi complex | 3.49 |
| HISTO_ZL.Contig1131.eannot.1960.final_new | KAE1 SGDID:S000001746 Putative glycoprotease | 3.48 |
| HISTO_ZU.Contig65.Fgenesh_histo.62.final_new | EXO1 SGDID:S000005559 5'-3' exonuclease and flap-endonuclease | 3.48 |
| HISTO_JG.Contig206.Fgenesh_Aspergillus.16.final_new | HXT3 SGDID:S000002753 Low affinity glucose transporter | 3.43 |
| HISTO_ZT.Contig1089.eannot.1671.final_new | WSC2 SGDID:S000005227 sensor-transducer of the stress-activated PKC1-MPK1 signaling pathway | 3.37 |
| HISTO_LG.Contig392.Fgenesh_histo.72.final_new | PFA3 SGDID:S000005270 Palmitoyltransferase for Vac8p | 3.36 |
| HISTO_DU.Contig190.Fgenesh_Aspergillus.72.final_new | CGDID:CAL0004562 Putative transporter; slightly similar to the Sit1p siderophore transporter | 3.34 |
| HISTO_DM.Contig936.eannot.1264.final_new | HSP31 SGDID:S000002941, Possible chaperone and cysteine protease | 3.33 |
| HISTO_ZL.Contig1161e.Fgenesh_histo.80.final_new | MUC1 SGDID:S000001458 GPI-anchored cell surface glycoprotein | 3.32 |
| HISTO_ZL.Contig1117.Fgenesh_histo.14.final_new | SNT1 CGDID:CAL0003554 NAD-independent histone deacetylase | 3.27 |
| HISTO_ZE.Contig158-snap.9.final_new | ERG25 SGDID:S000003292 C-4 methyl sterol oxidasemethyl utilized in ergosterol biosynthesis | 3.25 |
| HISTO_ZL.Contig1131.eannot.2134.final_new | RGT2 SGDID:S000002297 Plasma membrane glucose receptor | 3.25 |
| HISTO_LF.Contig359.eannot.1977.final_new | RGT2 SGDID:S000002297 Plasma membrane glucose receptor | 3.24 |
| HISTO_ZE.Contig158.eannot.1415.final_new | CYB2 SGDID:S000004518 Cytochrome b2 | 3.18 |
| HISTO_DM.Contig940.eannot.1255.final_new | TNA1 SGDID:S000003492High affinity nicotinic acid plasma membrane permease | 3.17 |
| HISTO_ZL.Contig1131.fgenesh_plus.131.final_new | ARO4 SGDID:S000000453 3-deoxy-D-arabino-heptulosonate-7-phosphate (DAHP) synthase | 3.17 |
| HISTO_GL.Contig59.eannot.1242.final_new | DPP1 SGDID:S000002692Diacylglycerol pyrophosphate (DGPP) phosphatase | 3.11 |
| HISTO_KF.Contig601.eannot.1274.final_new | SST2 SGDID:S000004444GTPase-activating protein for Gpa1p, regulates desensitization to alpha factor pheromone | 3.08 |
| HISTO_GL.Contig59.genewise.39.final_new | DBF4 SGDID:S000002459Regulatory subunit of Cdc7p-Dbf4p kinase complex | 3.07 |
| HISTO_HS.Contig68.genewise.187.final_new | AKR1 SGDID:S000002672  Palmitoyl transferase involved in protein palmitoylation | 3.05 |
