# Supplementary material for: Insertional mutagenesis enables cleistothecial formation in a non-mating strain of Histoplasma capsulatum
Source: BMC Microbiol. 2010 Feb 16;10:49. doi: 10.1186/1471-2180-10-49 (PMC2834667; doi:10.1186/1471-2180-10-49)
Supplement: Additional file 2 — Genes downregulated in UC26 vs G217B. This file contains a listing of all genes downregulated 3 fold or more in H. capsulatum strain UC26 compared to G217B. The data includes the H. capsulatum gene name, the gene annotation and the fold change. [file 1471-2180-10-49-S2.DOC]

### Additional file 2 – Genes downregulated in UC26 vs G217B

| ***H. capsulatum* gene names[42]:** | **Gene annotation[42]:** | **Fold change** |
| --- | --- | --- |
| HISTO_ZL.Contig1161e.Fgenesh_histo.33.final_new | MDL1 SGDID:S000004178 ABC  transporter, inner mitochondrial membrane | -26.96 |
| HISTO_ZT.Contig1128f.genewise.21.final_new | BGL22 CGDID:CAL0004607 Putative glucanase | -20.67 |
| HISTO_ZT.Contig1089.Fgenesh_Aspergillus.75.final_new | DER1 SGDID:S000000405 Endoplasmic reticulum membrane protein | -19.63 |
| HISTO_DY.Contig31.genewise.11.final_new | VBA2 SGDID:S000000497 Permease of basic amino acids in the vacuolar membrane | -16.91 |
| HISTO_ZT.Contig1129.Fgenesh_histo.4.final_new | ATG26 SGDID:S000004179 UDP-glucose:sterol glucosyltransferase | -16.02 |
| HISTO_ZT.Contig181.eannot.1612.final_new | PEP12 SGDID:S000005562 Target membrane receptor (t-SNARE) for vesicular intermediates | -13.08 |
| HISTO_ZL.Contig1131.eannot.2042.final_new | SSC1 SGDID:S000003806 Mitochondrial matrix ATPase involved in protein translocation and folding | -12.84 |
| HISTO_ZL.Contig1161c.Fgenesh_Aspergillus.21.final_new | TPO1 SGDID:S000003951 Polyamine transporter; catalyzes uptake of polyamines at alkaline pH and excretion at acidic pH | -11.71 |
| HISTO_GL.Contig286.fgenesh_plus.7.final_new | SDT1 SGDID:S000003192 Pyrimidine nucleotidase | -10.5 |
| HISTO_ZL.Contig1131.fgenesh_plus.131.final_new | ARO4 SGDID:S000000453 catalyzes the first step in aromatic amino acid biosynthesis | -9.86 |
| HISTO_LF.Contig359.eannot.2085.final_new | PIM1 SGDID:S000000118 protease involved in degradation of misfolded proteins in mitochondria | -9.31 |
| HISTO_ZT.Contig1089.Fgenesh_histo.158.final_new | RAD54 SGDID:S000003131 DNA-dependent ATPase | -8.99 |
| HISTO_GL.Contig59.eannot.1242.final_new | DPP1 SGDID:S000002692 Diacylglycerol pyrophosphate (DGPP) phosphatase | -8.96 |
| HISTO_ZL.Contig1161c.eannot.1502.final_new | TIF34 SGDID:S000004754 Subunit of translation initiation factor 3 | -8.34 |
| HISTO_ZL.Contig1158.eannot.1365.final_new | SEC18 SGDID:S000000284 ATPase required for the release of Sec17p during vacuole fusion and for ER to Golgi transport | -8.12 |
| HISTO_GI.Contig382.eannot.1251.final_new | FMO1 SGDID:S000001219 Flavin-containing monooxygenase | -8.04 |
| HISTO_HS.Contig68.eannot.2016.final_new | SGDID:S000002536 Fimbrin, actin-bundling protein | -7.87 |
| HISTO_LG.Contig392.Fgenesh_histo.72.final_new | PFA3 SGDID:S000005270 Palmitoyltransferase required for vacuolar membrane fusion | -7.42 |
| HISTO_DY.Contig31.eannot.1414.final_new | ERP4 SGDID:S000005542 involved in ER to Golgi transport" | -7.2 |
| HISTO_ZT.Contig174.eannot.1503.final_new | GYP7 SGDID:S000002393 GTPase-activating protein for yeast Rab family members | -7.14 |
| HISTO_ZT.Contig174.Fgenesh_histo.50.final_new | SKT5 SGDID:S000000157 Activator of Chs3p (chitin synthase III) | -6.92 |
| HISTO_HS.Contig68-snap.106.final_new | Aspergillus fumigatus: acetyltransferase, GNAT family family | -6.66 |
| HISTO_KF.Contig601.Fgenesh_histo.91.final_new | BPT1 SGDID:S000003938ABC type transmembrane transporter of MRP/CFTR family | -6.04 |
| HISTO_ZL.Contig1161d.Fgenesh_histo.38.final_new | ENO1 SGDID:S000003486 Enolase I | -5.88 |
| HISTO_ZU.Contig65.eannot.1956.final_new | SKP1 SGDID:S000002736 Kinetochore protein | -5.83 |
| HISTO_EA.Contig33.Fgenesh_histo.172.final_new | RPN3 SGDID:S000000823 subunit of the 26S proteasome lid | -5.71 |
| HISTO_ZZ.Contig127c.eannot.1578.final_new | VPS60 SGDID:S000002894 Cytoplasmic and vacuolar membrane protein involved in late endosome to vacuole transport | -5.66 |
| HISTO_HS.Contig68.Fgenesh_Neurospora.222.final_new | RPL37B SGDID:S000002908 Protein component of the large (60S) ribosomal subunit | -5.58 |
| HISTO_DM.Contig933.Fgenesh_histo.4.final_new | RPN2 SGDID:S000001337 Subunit of the 26S proteasome | -5.53 |
| HISTO_LF.Contig359.eannot.2034.final_new | PPS1 SGDID:S000000480 Protein phosphatase with specificity for serine, threonine, and tyrosine residues | -5.51 |
| HISTO_ZL.Contig1131.eannot.2001.final_new | ACT1 CGDID:CAL0001571 Actin | -5.36 |
| HISTO_FE.Contig19.Fgenesh_histo.134.final_new | Aspergillus nidulans: heat shock protein 90 | -5.34 |
| HISTO_DM.Contig933.eannot.1625.final_new | ALD2 SGDID:S000004780 Cytoplasmic aldehyde dehydrogenase | -5.24 |
| HISTO_ZZ.Contig127c.Fgenesh_histo.37.final_new | VTI1 SGDID:S000004810 Protein involved in cis-Golgi membrane traffic | -5.21 |
| HISTO_ZZ.Contig127c-snap.39.final_new | MNN11 SGDID:S000003719 Subunit of a Golgi mannosyltransferase complex | -5.15 |
| HISTO_DM.Contig936.eannot.1239.final_new | ARD1 SGDID:S000001055 Subunit of the N-terminal acetyltransferase NatA | -5.13 |
| HISTO_GL.Contig233.eannot.1168.final_new | CYC7 SGDID:S000000765 Cytochrome c isoform2 | -5.11 |
| HISTO_ZT.Contig1089.Fgenesh_Aspergillus.107.final_new | ERG26 SGDID:S000002969 C-3 sterol dehydrogenase | -5.06 |
| HISTO_DY.Contig31.Fgenesh_histo.45.final_new | VPS1 SGDID:S000001709 GTPase required for vacuolar protein sorting | -4.99 |
| HISTO_ZT.Contig1089.eannot.1634.final_new | ACS1 SGDID:S000000050 Acetyl-coA synthetase isoform | -4.9 |
| HISTO_HS.Contig68.Fgenesh_histo.109.final_new | CDC9 SGDID:S000002323 DNA ligase | -4.87 |
| HISTO_ZZ.Contig127c.eannot.1593.final_new | RPS6A SGDID:S000006011 40S ribosomal subunit | -4.86 |
| ISTO_ZL.Contig1161e.Fgenesh_Aspergillus.88.final_new | UBI4 SGDID:S000003962 Ubiquitin | -4.8 |
| HISTO_ZL.Contig1131.eannot.1973.final_new | CAM1 SGDID:S000005969 Translational cofactor elongation factor-1 gamma | -4.74 |
| HISTO_ZT.Contig1141.Fgenesh_histo.3.final_new | ERG5 SGDID:S000004617 C-22 sterol desaturase | -4.74 |
| HISTO_LF.Contig359.eannot.2091.final_new | RPS15 SGDID:S000005400 Protein component of the small (40S) ribosomal subunit | -4.69 |
| HISTO_ZL.Contig1131-snap.36.final_new | MSS1 SGDID:S000004625 Mitochondrial protein | -4.6 |
| HISTO_ZL.Contig1131.eannot.2108.final_new | APL1 SGDID:S000003765 Beta-adaptin, large subunit of the clathrin associated protein complex | -4.6 |
| HISTO_HS.Contig68.eannot.2113.final_new | IMP3 SGDID:S000001191 Component of the SSU processome | -4.43 |
| HISTO_HS.Contig68.Fgenesh_histo.257.final_new | DBP5 SGDID:S000005572 RNA helicase of the DEAD-box family involved in mRNA export from the nucleus | -4.38 |
| HISTO_ZU.Contig65.fgenesh_plus.46.final_new | FUR1 SGDID:S000001170 Uracil phosphoribosyltransferase | -4.37 |
| HISTO_ZL.Contig658.eannot.1266.final_new | BIM1 SGDID:S000000818 Microtubule-binding protein | -4.35 |
| HISTO_LG.Contig392.eannot.1792.final_new | SAT4 SGDID:S000000601 Ser/Thr protein kinase involved in salt tolerance | -4.34 |
| HISTO_DU.Contig190-snap.24.final_new | Aspergillus fumigatus: acetyltransferase, GNAT family | -4.25 |
| HISTO_DY.Contig31.Fgenesh_histo.94.final_new | NPY1 SGDID:S000003035 NADH diphosphatase | -4.25 |
| HISTO_ZU.Contig65.eannot.2038.final_new | SES1 SGDID:S000002430 Cytosolic seryl-tRNA synthetase | -4.25 |
| HISTO_LF.Contig359.eannot.2026.final_new | RIO2 SGDID:S000005151 kinase involved in processing 20S pre-rRNA | -4.22 |
| HISTO_GY.Contig460.genewise.7.final_new | ATG7 SGDID:S000001214 Autophagy-related protein | -4.16 |
| HISTO_LF.Contig359.Fgenesh_histo.315.final_new | DCP2 SGDID:S000005062 removes the 5' cap structure from mRNAs prior to their degradation | -4.08 |
| HISTO_EA.Contig33.fgenesh_plus.37.final_new | SLA2 SGDID:S000005187 Transmembrane actin-binding protein involved in membrane cytoskeleton assembly and cell polarization | -4.04 |
| HISTO_HS.Contig68.Fgenesh_Aspergillus.237.final_new | GAS5 SGDID:S000005390 Putative 1,3-beta-glucanosyltransferase | -3.96 |
| HISTO_ZZ.Contig127b-snap.45.final_new | NAR1 SGDID:S000005184 iron-sulfur (FeS) protein assembly | -3.81 |
| HISTO_ZU.Contig65.eannot.1967.final_new | BUD31 SGDID:S000000659 Protein involved in bud-site selection | -3.77 |
| HISTO_ZL.Contig1158-snap.1.final_new | TPO3 SGDID:S000006360 Polyamine transport protein specific for spermine | -3.74 |
| HISTO_ZU.Contig2.fgenesh_plus.5.final_new | ADH5 SGDID:S000000349 Alcohol dehydrogenase isoenzyme V | -3.74 |
| HISTO_DM.Contig933.eannot.1689.final_new | SUB2 SGDID:S000002242 DEAD-box RNA helicase | -3.71 |
| HISTO_ZT.Contig1089.Fgenesh_histo.198.final_new | GAR1 SGDID:S00000113 Protein involved in the modification and cleavage of the 18S pre-rRNA | -3.7 |
| HISTO_ZT.Contig181.eannot.1695.final_new | POL2 SGDID:S000005206 Catalytic subunit of DNA polymerase epsilon | -3.69 |
| HISTO_ZL.Contig1161c.eannot.1440.final_new | IDP1 SGDID:S000002224 Mitochondrial NADP-specific isocitrate dehydrogenase | -3.68 |
| HISTO_ZT.Contig181.eannot.1730.final_new | UFD2 SGDID:S000002349 Ubiquitin chain assembly factor (E4) | -3.68 |
| HISTO_ZL.Contig1161b.Fgenesh_Aspergillus.34.final_new | RPA43 SGDID:S000005867 RNA polymerase I subunit A43 | -3.67 |
| HISTO_ZL.Contig1161d.eannot.1501.final_new | PUP3 SGDID:S000000896 Beta subunit of the 20S proteasome | -3.51 |
| HISTO_GY.Contig471.eannot.1315.final_new | BTS1 SGDID:S000005990 Geranylgeranyl diphosphate synthase | -3.46 |
| HISTO_ZL.Contig1161d.Fgenesh_histo.85.final_new | NAT2 SGDID:S000003379 N alpha-acetyl-transferase | -3.39 |
| HISTO_GY.Contig460.Fgenesh_Aspergillus.204.final_new | SPT16 SGDID:S000003175 Subunit of the heterodimeric FACT complex | -3.38 |
| HISTO_EA.Contig33.eannot.1591.final_new | UBC4 SGDID:S000000286 Ubiquitin-conjugating enzyme | -3.32 |
| HISTO_ZL.Contig1161a.Fgenesh_histo.2.final_new | BOI2 SGDID:S000000916 Protein implicated in polar growth | -3.32 |
| HISTO_GY.Contig460.fgenesh_plus.97.final_new | ATG3 SGDID:S000005290 Protein involved in autophagy | -3.25 |
| HISTO_HF.Contig5.eannot.1028.final_new | MES1 SGDID:S000003496 Methionyl-tRNA synthetase | -3.24 |
| HISTO_FX.Contig167.eannot.1298.final_new | Deoxycytidyl transferase; involved in repair of abasic sites in damaged DNA | -3.22 |
| HISTO_ZZ.Contig127a.fgenesh_plus.60.final_new | PRP31 SGDID:S000003323 Splicing factor | -3.22 |
| HISTO_ZT.Contig181.fgenesh_plus.113.final_new | CCT6 SGDID:S000002596 Subunit of the cytosolic chaperonin Cct ring complex | -3.2 |
| HISTO_LF.Contig359-snap.57.final_new | CDC48 SGDID:S000002284 ATPase in ER, nuclear membrane and cytosol | -3.19 |
| HISTO_ZU.Contig65.eannot.2104.final_new | RPS20 SGDID:S000001007 Protein component of the small (40S) ribosomal subunit | -3.19 |
| HISTO_ZT.Contig174.Fgenesh_histo.17.final_new | PUF3 SGDID:S000003936 Protein that regulates degradation of specific mRNAs | -3.16 |
| HISTO_DY.Contig31.eannot.1380.final_new | COX6 SGDID:S000001093 Subunit VI of cytochrome c oxidase | -3.14 |
| HISTO_LF.Contig359.eannot.1926.final_new | PDI1 SGDID:S000000548 Protein disulfide isomerase | -3.14 |
| HISTO_LF.Contig359-snap.93.final_new | VPS16 SGDID:S000005966 Subunit of the homotypic vacuole fusion and vacuole protein sorting (HOPS) complex | -3.11 |
| HISTO_LF.Contig359.Fgenesh_histo.281.final_new | PTC1 SGDID:S000002164 Type 2C protein phosphatase; inactivates the osmosensing MAPK cascade | -3.11 |
| HISTO_ZU.Contig65.eannot.2017.final_new | SUP45 SGDID:S000000347 Polypeptide release factor involved in translation termination | -3.1 |
| HISTO_ZZ.Contig127c.eannot.1516.final_new | ISA1 SGDID:S000003950, Chr XII from 87402-88154, Verified ORF, "Mitochondrial matrix protein involved in biogenesis of the iron-sulfur (Fe/S) cluster of Fe/S proteins | -3.08 |
| HISTO_EA.Contig33.eannot.1674.final_new | CDS1 SGDID:S000000233 Phosphatidate cytidylyltransferase | -3.06 |
| HISTO_JG.Contig206.eannot.1483.final_new | HWP1 CGDID:CAL0003753 Hyphal cell wall protein | -3.04 |
| HISTO_LF.Contig359.fgenesh_plus.105.final_new | CYS3 SGDID:S000000010 Cystathionine gamma-lyase | -3.03 |
